# Does symmetry preclude the evolution of senescence? A comment on Pen and Flatt 2021

**DOI:** 10.1098/rspb.2022.1101

**Published:** 2023-01-25

**Authors:** Charlotte de Vries, E. Yagmur Erten, Hanna Kokko

**Affiliations:** ^1^ Department of Evolutionary Biology and Environmental Studies, University of Zurich, Zurich, Switzerland; ^2^ Department of Biological and Environmental Science, University of Jyväskylä, Jyväskylä, Finland; ^3^ Institute for Biodiversity and Ecosystem Dynamics, University of Amsterdam, Amsterdam, The Netherlands; ^4^ Konrad Lorenz Institute of Ethology, University of Veterinary Medicine, Vienna, Austria; ^5^ Faculty of Biological and Environmental Sciences, University of Helsinki, Helsinki, Finland

Patterns of senescence across the tree of life remain poorly understood and a clearly important task is to identify the minimal conditions for senescence to occur at all. Senescence refers to changes in phenotype that cause an increase in mortality rate, or decrease in fertility rate, with age. Starting with Weismann in 1882, it has generally been argued that some type of asymmetry between parent and offspring is a prerequisite for old individuals to show declining performance [1–4]. The intuitive role of asymmetries should, however, be subject to mathematical scrutiny. Highly interestingly, recent work has highlighted results that counter the above intuition: Pen & Flatt (hereafter PF) recently reported that senescence *can* evolve in an organism that reproduces via symmetrical division, concluding that ‘[ … ] the evolution of senescence might, therefore, be inevitable [ … ]’ [5, p. 8].

Here we show that the ‘symmetric’ division of PF does not successfully remove all asymmetries between the two individuals that exist after reproduction. However, envisaging a fully symmetrical scenario is not straightforward either: assigning ‘age’ and the labels ‘parent’ and ‘offspring’ to individuals (or cells) presents non-trivial challenges. As a whole, this highlights that seeking for minimal conditions under which senescence can occur is a difficult task as it operates in a definitional minefield.

We will first briefly introduce the model structure of PF. The life cycle starts with a single cell, which doubles once during development to form a two-celled adult individual. In line with PF, we will use the terms ‘parent’ and ‘offspring’ to refer to two-celled individuals. Only one of the two cells in the parent gives rise to any one focal offspring (although both cells are capable of doing so, independently), and reproduction proceeds through a single-celled state before the doubling required to become a mature individual. We refer to the individual cells as ‘mother cell’ and its ‘daughter cell’. Importantly for what follows, PF assign an age of 1 to the offspring, while the parent’s age (an integer *a* ≥ 1) increases by 1 (note that the mother cell still exists within the two-celled parent after reproduction).

Senescence occurs in PF’s model because damage accumulates within each cell, at a rate that can be counteracted by repair processes. Specifically, mutation yields a supply of deleterious alleles, which are expressed at specific damage levels, and this yields mortality rates that change with age. PF also assume that cell division allows the mother cell to transmit half of its damage to the daughter cell, and the offspring starts its development with inherited damage (figure 1*a*). The mother cell thereafter keeps being part of the parent, who has aged by 1 unit chronologically, while half of it (one cell out of two) has experienced rejuvenation.

**Figure 1.**
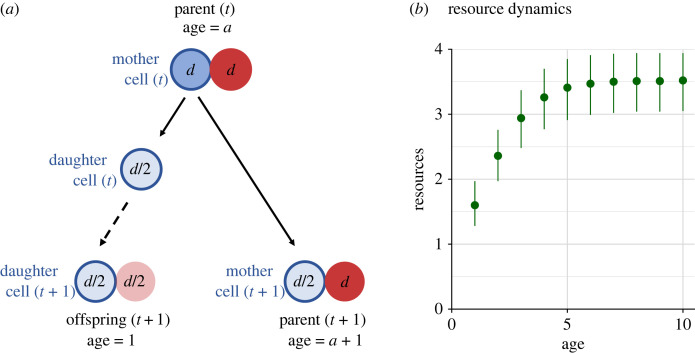
(*a*) Life cycle of the model of Pen & Flatt [5]. ‘Parent’ and ‘offspring’ are two-celled adult individuals; for clarity, we let ‘daughter cell’ refer to the single-celled stage before development, and ‘mother cell’ to the ancestor cell of this daughter. Solid and dashed lines refer to events during reproduction and to development from the single-celled stage to an adult, respectively. Colours indicate damage level (darker for more damage) and cell type (blue with solid border for type 1, red without border for type 2; note, however, that motherhood is possible for either type, and type 1 reproduces in the example for illustration only). Importantly, during reproduction, the mother experiences rejuvenation (damage halves from *d* to *d*/2), since the daughter cell receives half of its damage. An asymmetry between parent and offspring arises during development: the daughter cell copies its damage while developing into a two-celled adult, resulting in an offspring that is, on average, more rejuvenated than its parent; the parent rejuvenates to a lesser extent as it also has a cell (here, the red cell) unimpacted by reproduction. (*b*) Example of a resource curve using the parameters from example 1 in figure 2*b*,*e*, parameters listed below figure 2.

The subsequent developmental process that leads from the daughter cell to an adult offspring is somewhat ambiguously described by PF: the daughter cell ‘develops into an adult by doubling once, thereby splitting resources and damage equally between itself (henceforth designated type 1) and its daughter cell (designated type 2)’ [5, p. 6]. Here, their language of ‘splitting’ is problematic: to us, this verb suggests that each of the two cells of a fully developed offspring contain half of the damage of the original daughter cell, but an inspection of the simulation code of PF shows this not to be the correct interpretation. Instead, the offspring is formed by simply duplicating the original daughter cell, leading to damage levels as depicted in figure 1*a*.

Despite PF hoping to have created a situation that lacks asymmetries, reproduction introduces an asymmetry in accumulated damage between the parent and offspring individuals. To understand why, let us look at a case where each cell of the parent individual has the population average cell-specific damage level, *d*, before reproduction (figure 1*a*). Since the parent cell transfers half of its damage to the offspring cell during reproduction, the parent individual will have a total damage level of 3*d*/2 after reproduction (*d*/2 in the cell that has just reproduced and *d* in the other one, assuming only one of the cells reproduces). The offspring cell will inherit *d*/2 of the damage of its parent and after development, the adult offspring will have a total damage of *d* (based on the implementation in simulation code of Pen & Flatt [5]; *d*/2 based on the division rules described in their article). Because the offspring are also assigned an age of 1 while the parent has a higher age, this asymmetry creates a correlation between age and damage level. Of course, occasionally both cells of the parent individual will reproduce at the same time which would lead to a more equal damage distribution, on average.

The division of resources between mother and daughter cell introduces a second parent–offspring asymmetry. During reproduction, a proportion of resources are given to the daughter cell, according to the parent cell’s investment into reproduction. In practice, this means that daughter cells start off with less resources than their mother cell, leading to a resource function that increases with age (figure 1*b*).

The issues described above highlight the difficulty of defining symmetric division in the context of a multicellular life cycle. PF aimed for a situation in which the mother cell (measured after reproduction) and the daughter cell (which develops into a ‘new’ individual) have equivalent properties. However, given that the resource and damage levels of the partner cell differ in a systematic way between parent and offspring, the subsequent demography is different too; to us, this is best conceptualized as parent–offspring asymmetries existing in the model.

Note, however, that while average individual phenotypes change with chronological age, only a subset of changes also imply senescence. Depending on the damage distribution, which itself is a function of multiple model parameters, it is possible to evolve a diverse range of mortality trajectories, including those in which the mortality rate steadily increases with age (senescence), as well as some others in which it does not (figure 2). As described in PF, different mortality trajectories arise owing to some damage levels being rare in the population, leading to weaker selection against the fixation of deleterious alleles that are expressed in those damage levels, compared to selection against the alleles expressed in more common damage levels. Therefore, whereas parent–offspring asymmetries in PF's model can associate with the evolution of senescence, they do not exclude the evolution of negative or negligible senescence; the evolution of senescence is not inevitable.

**Figure 2.**
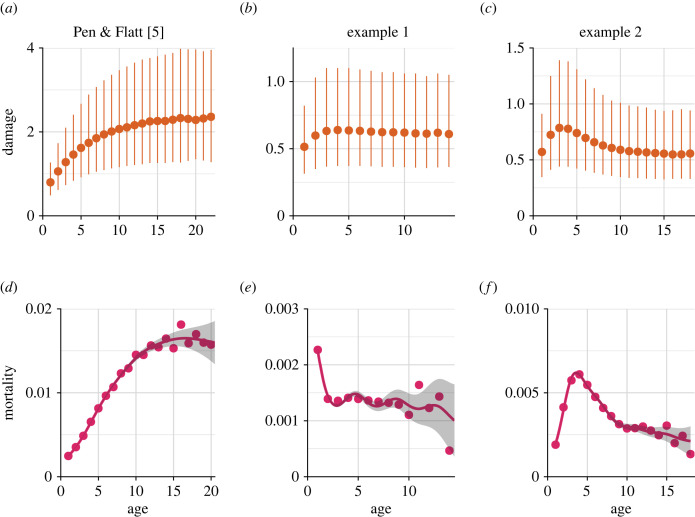
Three examples of evolved age-specific mortality curves. The three scenarios differ in parameterization, which primarily affects the resource accumulation (via shape parameters *h* and *f*_max_), rate of increase in reproduction probability with resources (*b*), modularity (an individual’s ability to survive when one of its cells die), and damage-induced cellular mortality (which is relatively small when *D* = 1000, and vanishes when *D* = ∞, allowing us to focus on the effect of deleterious alleles). For all three scenarios, *x*-axes show age in all panels; *y*-axes show damage level on top, and mortality rate on the bottom. (*a*,*d*) The scenario in figure 1 of Pen & Flatt [5]. (*b*,*e*) An example with modularity, slower increase of resources, and higher *b*, and (*c*,*f*) an example with no modularity, slower increase of resources and higher *b* than figure 1 of PF. Parameters that differ between scenarios are (using the notation of Pen & Flatt [5]), (*a*,*d*): *f*_max_ = 6, *h* = 0.3, *b* = 0.1, *D* = 1000, *m*_2_ = 0.05, *d*_max_ = 6; (*b*,*e*): *f*_max_ = 6, *h* = 1.0, *b* = 0.8, *D* = ∞, *m*_2_ = 0.08, *d*_max_ = 4; (*c*,*f*): *f*_max_ = 6 *h* = 2.0, *b* = 2, *D* = ∞, *m*_2_ = 0.08, *d*_max_ = 4. Other parameters are as listed in table 1 in [5]. Data are collected during the last 25 timesteps and averaged across timesteps. Code for all figures can be found at https://github.com/Lotte-biology/PF-symmetry-senescence.

Even if, in a particular case (such as the PF model), senescence can be linked to parent–offspring asymmetries, this does not yet counteract the suspicion of PF that senescence can evolve in the absence of such asymmetries; instead it simply means that a fully symmetric situation remains unexplored so far in their model. Such a setting can be created within the two-cell framework of PF (figure 3*a*). In this scenario, both cells of the parent adult divide and the resulting products stay together to form two new adults that are identical to each other in terms of damage level (as well as resources; not shown in figure 3 for simplicity). This process ensures symmetry between the two products of reproduction. Although this scenario of symmetric division can still lead to the evolution of senescence under certain parameterizations (results not shown), it creates a conceptual problem, which we turn to below.

**Figure 3.**
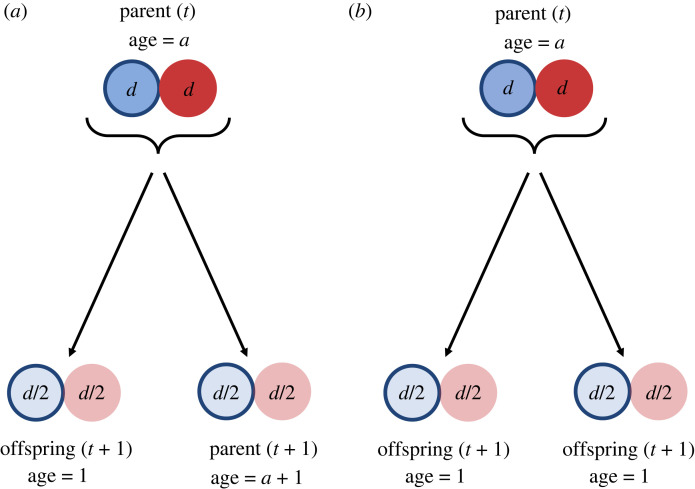
(*a*) A hypothetical model of reproduction that results in two identical adult individuals with the same damage level, with their ages defined as in Pen & Flatt [5]. (*b*) The same model as in (*a*) with age defined as age-since-division (chronological age), and no parent surviving the division.

If reproduction creates two identical adults, which one should be labelled as the parent (with age *A*_parent_ = *a* + 1) and which one as the offspring (with age *A*_offspring_ = 1)? Can we justify arbitrarily labelling one of the individuals as the older parent and the other one as the younger offspring if they are genuinely identical (as in figure 3*a*)? It appears difficult to assign different chronological ages to the two identical end products of reproduction; yet, any statements of senescence require there to be individuals that differ in their chronological ages.

This conceptual difficulty arises partly because PF model parent–offspring symmetry in the context of a multicellular life cycle that passes through a unicellular life stage, whereas statements about symmetry precluding the evolution of senescence are generally made either in a unicellular context [6], or in the context of multicellular organisms that reproduce through symmetrical fission (e.g. [7]). The question of how to define the boundaries of one ‘life’ in unicellular and/or modular organisms has plagued the field of senescence from the beginning [4]. Present-day microbiologists distinguish between chronological lifespan (how long a cell can remain viable without dividing), and replicative lifespan (number of divisions before the lineage dies [8,9]). When studying whether symmetry precludes the evolution of senescence, one should, therefore, specify if senescence is defined as senescence between divisions (‘chronological senescence’, phenotypic changes and associated increase in mortality rate between two cell divisions), or lineage senescence (progressive deterioration of and associated increase in mortality of a cell population over time).

Translating age and senescence concepts described above to multicellular organisms is non-trivial. One can argue that development from a single cell to a two-celled adult marks the beginning of a lifespan, and this allows PF to call one of the individuals after reproduction as the ‘offspring’, even though offspring and parent are indistinguishable. We maintain that some degree of asymmetry is required to be able to define one individual as parent of older age and the other one as offspring of younger age, as also suggested by Ackermann *et al.* [6]. Therefore, if the end products of reproduction are identical, we suggest defining the multicellular organism before reproduction as the older parent and the products of reproduction as the offspring (as in figure 3*b*). This scenario is analogous to symmetry in a unicellular context, where senescence between cell divisions can evolve if the lineage is protected from extinction via dilution of damage during reproduction (unless damage, or a part of it, concerns heritable damage that is strictly and equally passed on to the offspring, e.g. damage in genetic material). That is, this type of symmetry precludes the evolution of lineage senescence but it does not preclude the evolution of chronological senescence (see also discussion in [6]).

So does symmetry preclude the evolution of senescence? We argue that PF do not fully answer this question for several reasons beyond the asymmetries in their original model. PF modelled a process suggested to operate in unicellular organisms in a multicellular context, which allowed a definition of ‘offspring’ and ‘age’ that can be problematic to apply in unicellular contexts. As they did not specify whether they are interested in lineage or chronological senescence (or both), it may be useful to be aware of certain limitations of the scope of their work: they showed chronological senescence can evolve under a multicellular definition of age, but did not address lineage senescence. The issue is not unique to their work. It is often ambiguous what is meant with the statement ‘symmetry precludes the evolution of senescence’ in the literature; our interpretation is that the statement usually pertains to lineage senescence. Supporting this interpretation is the fact that [6] previously showed that dilution allows a symmetrically dividing lineage to persist despite evolving chronological senescence, although it was not the main focus of their paper.

While we have been somewhat critical of the interpretation of PF, it is useful to remember its virtues. For instance, their flexible model structure allows changing whether an individual will die after one cell dies, or whether regeneration is possible. This increased modularity of the individuals in the model makes it possible to find scenarios where negative senescence evolves (figure 2*f*). Thus, despite the underlying asymmetries and conceptual difficulties regarding parent–offspring symmetry and age assignment, the model by Pen & Flatt [5] serves as a valuable framework to study the underlying reasons why species, sometimes even when they are relatively closely related, differ in their senescence patterns [10].

## Data Availability

All code used in this paper was obtained from https://github.com/idopen/asymmetry_and_ageing. Edited code files used to create figure 2 can be found at https://github.com/Lotte-biology/PF-symmetry-senescence; simulation data at https://figshare.com/articles/dataset/Simulation_data_and_code/19232535.
